# Functional pH-Sensitive Film Containing Purple Sweet Potato Anthocyanins for Pork Freshness Monitoring and Cherry Preservation

**DOI:** 10.3390/foods13050736

**Published:** 2024-02-28

**Authors:** Fahui Ke, Duanwu Liu, Juanjuan Qin, Min Yang

**Affiliations:** College of Science, Gansu Agricultural University, Lanzhou 730070, China; kefh534@163.com (F.K.); liurw@gsau.edu.cn (D.L.); isqinjj@163.com (J.Q.)

**Keywords:** pH-sensitive packaging film, purple sweet potato extract, polyvinyl alcohol, sodium alginate, sodium carboxymethyl cellulose, biodegradability

## Abstract

An antioxidative and pH-sensitive multifunctional film, incorporating anthocyanin-rich purple sweet potato extract (PPE) was fabricated from polyvinyl alcohol (PVA) and sodium alginate (SA)/sodium carboxymethyl cellulose (CMC-Na). The film was composed of 6:4 PVA:SA/CMC-Na (mass ratio, SA:CMC-Na at 1:1) with added PPE, and changed color with changes in pH, and also had useful UV-blocking, antioxidant, mechanical, and water vapor barrier properties, which enable its use as a food coating film. In addition, the incorporation of 300 mg PPE increased the biodegradability of the film in soil from 52.47 ± 1.12% to 64.29 ± 1.75% at 17 days. The pH sensitivity of the film enabled its successful use for the evaluation of pork freshness. Cherries coated with the film had an extended shelf life from 3–4 to 7–9 days, during storage at 25 °C. Consequently, the multifunctional film can be applied to packaging for real-time pH/freshness monitoring and for effectively preserving the freshness of meat and fruit.

## 1. Introduction

In recent years, the increasing consumer demand for improved food quality and safety has stimulated the development of functional food packaging. Conventional plastic film packaging materials perform only a simple barrier function and are non-biodegradable, so they cause waste disposal problems. Consequently, there has been considerable research interest in packaging films based on biodegradable materials, such as hydrophilic synthetic polymers, polysaccharides, phospholipids, and proteins [1]. Polyvinyl alcohol (PVA) has good film forming properties, biodegradability, and ready availability, as well as good barrier and mechanical properties and being safe and non-toxic, so it has been widely used in food packaging materials as one of the matrices [2]. In order to improve the barrier properties of pure PVA films, polysaccharides and/or proteins were introduced into the matrix which could increase the average length of the polymer chains through hydrogen bonding and electrostatic interactions to form network structures [3]. Polysaccharides such as sodium alginate (SA) [4], sodium carboxymethyl cellulose (CMC-Na) [5], cashew gum [6], and agarose [7] have been used to improve PVA films. 

SA is a natural polymer extracted from brown algae, containing abundant hydroxyl and carboxylic acid groups with excellent mechanical and film forming properties, which has been widely applied in food packaging films, combined with PVA [4]. The incorporation of SA enhanced the tensile strength (TS) and elongation at break (EAB) of PVA films, as well as increasing the density of the film structure and decreasing water vapor permeability [8]. PVA/SA films also had excellent mechanical properties and a high liquid absorption [8]. CMC-Na is comparable with SA for film formation and thickening, and is rich in hydroxyl groups, which interact with PVA to improve the water vapor barrier properties of films through hydrogen bonding [5]. For example, PVA/CMC-Na films are flexible and have a high tensile strength (TS) of 52.59 MPa and an EAB of 34.16% [9]. However, there were few studies on the properties of the films based on PVA/PVA/SA/CMC-Na ternary matrices.

Versatile biodegradable packaging films with functions such as ultraviolet blocking, antioxidation, and antibacterial properties, as well as sensing capabilities have attracted increasing research interest [2]. For example, pH-sensing films can act as food freshness indicators and food spoilage detectors [10]. They function during food transportation, storage, and sale by protecting food from contamination, enhancing its quality and prolonging its shelf-life [11]. The pH indicator system for food packaging consists of two components: the pH-sensing chromogenic agent and the support (film forming) material [2]. Synthetic pH indicators, such as bromocresol green, methyl red, cresol red, and bromomethyl blue change color in response to changes in the pH. However, they are toxic and carcinogenic and therefore cannot be used in contact with foods [12], so natural, non-toxic pH indicators must be used in functional packaging films [13]. Anthocyanins are the most commonly used natural pH indicators, because of their strong antioxidant and antibacterial properties and ready availability from vegetables, fruits, and flowers [14]. Various pH-sensitive films have been successfully prepared based on anthocyanins extracted from purple sweet potato peel [15], Roselle flowers [16], purple cabbage [7], and mulberries [17]. 

Purple sweet potato anthocyanins have high thermal stability and photostability and an excellent color response to pH changes, because of the abundance of the monoacylated and diacylated forms of cyanidin, resulting in a great application potential in pH-sensing packaging [18]. It has been proved that SA has a high affinity for anthocyanins, through electrostatic interactions and hydrogen bonding, while the positive charge on the anthocyanin molecule interacts with the carboxyl group of CMC-Na to reduce the migration of anthocyanins in the film and improve pH sensitivity [19,20]. PVA/CMC-Na/red cabbage anthocyanin films have a high pH sensitivity, TS, EAB, and water absorption [9]. Therefore, it was hypothesized that a combination of SA and CMC-Na may enhance the pH-sensitivity and stability of anthocyanins. However, there were few reports on pH indicating films made from a combination of PVA, SA, and CMC-Na with purple sweet potato anthocyanins as the pH indicator.

This study aimed to develop a versatile pH-indicating packaging film with antioxidant and ultraviolet blocking properties to monitor pork freshness and increase the shelf-life of cherries. Films were made by a one-pot method from PVA, SA, and CMC-Na, with purple sweet potato extract (PPE) as the pH indicator. The physicochemical and barrier properties of the films were evaluated, as well as their pH-sensitivity, antioxidant properties, and biodegradability. The preparation process of the films was simple, environmentally friendly, and cost-effective. The findings provide a theoretical basis for the development and application of films based on PVA/SA/CMC-Na on meat or fruit freshness monitoring and preservation. 

## 2. Materials and Methods

### 2.1. Materials

Polyvinyl alcohol (PVA, viscosity of 45.0–55.0 mPa·s), sodium alginate (SA, purity of 90%, M/G of 2:1), sodium carboxymethyl cellulose (CMC-Na, viscosity of 3000–5000 mPa·s), 2,2-diphenyl-1-picrylhydrazyl (DPPH), and 2,2-diazobis-(3-ethylbenzoxoline-6-sulfonic acid) diamine salt (ABTS) were purchased from Macklin Biochemical Co., Ltd. (Shanghai, China). Purple sweet potatoes, cherries, and fresh pork were purchased from the local supermarket (Lanzhou, China). Glycerol was obtained from Tianjin Baishi Chemical Co., Ltd. (Tianjin, China). Other reagents used in the article were of analytical level and acquired from Shanghai Yuanye Bio-Technology Co., Ltd. (Shanghai, China).

### 2.2. Extraction of Anthocyanins from Purple Sweet Potato and Their pH-Sensitivity

Anthocyanins were extracted from purple sweet potato based on the method of Zhao et al., with slight modifications [15]. Briefly, the purple sweet potatoes were cleaned with distilled water and cut into strips. The strips were dried and smashed at 40 °C in the dark to obtain purple sweet potato powder. The powder was blended with a 70% ethanol aqueous solution (1:10, *w*/*v*) for 4 h at 40 °C. The solution was then filtered to remove residues. The ethanol in the filtrate was removed using a rotary evaporator. Finally, purple sweet potato extract (PPE) rich in anthocyanins was dried using a vacuum freeze dryer (Beijing Bo Medical Kang Experimental Instrument Co., Ltd., Beijing, China) and stored in brown vials at 4 °C. The content of anthocyanin in PPE was 1.36 ± 0.06 mg/g determined using the pH differential method. 

The PPE solution was prepared by dissolving 40 mg PPE in 10 mL distilled water, the pH of which was adjusted between 2.0 and 13.0 using HCl (0.1 M) or NaOH (0.1 M). A UV–VIS spectrophotometer (UV–1780, Shimadzu Instrument, Co., Ltd., Tokyo, Japan) was used to record the spectrum of PPE solution across wavelengths ranging from 450 nm to 750 nm [15].

### 2.3. Film Preparation

The films were fabricated using the solution casting technique based on the following reference, with minor modifications [3]. PVA, SA, and CMC-Na with a total mass of 5 g were dissolved in 100 mL of deionized water, the mass ratio of which is shown in Table 1. After being magnetically stirred at 50 °C for 6 h, 1 g glycerin was added to the mixture and stirred for 1 h at the same conditions. Subsequently, the 10 g mixture was poured into a Petri dish (d = 90 mm) and dried for 6 h at 45 °C. Films were labeled based on the mass ratios of PVA to SA/CMC-Na as PSC-10:0, PSC-9:1, PSC-8:2, PSC-7:3, and PSC-6:4. For pH-sensitive films’ preparation, 0 mg, 300 mg, 500 mg, and 700 mg of PPE was added to the PSC-6:4 mixture and stirred at 50 °C for 2 h. The films were prepared based on the method above, and were named PSC-PPE-300, PSC-PPE-500, and PSC-PPE-700. All films were peeled from the Petri dishes after they were dried, and were stored at 4 °C.

### 2.4. Structural Characterization of Films

#### 2.4.1. Fourier Transform Infrared (FTIR) Spectroscopy Analysis

FTIR spectra of films were recorded using a Nicolet iS50 FTIR spectrometer (Thermo Fisher Scientific, Waltham, MA, USA) with an attenuated total reflection (ATR) mode cell. Measurements spanned a wavelength range of 500–4000 cm^−1^, were scanned 32 times, and had a resolution of 4 cm^−1^ [21].

#### 2.4.2. X-ray Diffraction (XRD) Analysis

XRD spectra of films were carried out with an XD3 X-ray polycrystal diffractometer (Beijing Purkinje General Instrument Co., Ltd., Beijing, China) under Cu Kα radiation in a continuous mode. Specific parameters were a scanning range from 10° to 80° (2θ), voltage of 40 kV, current of 30 mA, step size of 0.02°, and scanning speed of 5 °/min [22]. 

#### 2.4.3. Scanning Electron Microscopy (SEM) Analysis

The cross-sections, morphologies, and surface topographies of the films were observed using an S–3400N SEM (Hitachi Instruments Co., Ltd., Tokyo, Japan) operating at 5 kV with 1000× and 2000× magnification, according to Reference [23].

### 2.5. Physical Properties of Films

#### 2.5.1. Physical Appearance and Color

A smartphone camera was used to photograph the surface of the films. The color parameter of the films (L*—lightness, a*—redness–greenness, b*—yellowness–blueness) was analyzed using a CS–200 precision colorimeter (Hangzhou color spectrum Technology Co., Ltd., Hangzhou, China) [24]. The total color difference (Δ*E*) was calculated using Equation (1):

(1) $$\Delta E=\sqrt{\left({\Delta L}^{2}+{\Delta a}^{2}+{\Delta b}^{2}\right)}$$

where Δ*L* = *L* − L*; Δ*a* = *a* − a*; Δ*b* = *b* − b*; *L* of 88.02; *a* of −3.05; and *b* of −2.99 were the color parameters of the standard white plate; L*, a*, and b* were the parameters of PSC or PSC-PPE films.

#### 2.5.2. pH-Sensitivity

The films were completely immersed in varied phosphate-buffered solutions (pH ranging from 2.0 to 13.0) for 3 min. Subsequently, the film was instantly taken out and put on waterproof paper. The appearance of color variation was captured with a smartphone camera (iPhone 13, with 12 million pixels) [25].

#### 2.5.3. Thickness

The thickness values of the films were evaluated by measuring five random positions of the films using a handheld digital micrometer (Shengtaixin Electronic Technology Co., Ltd., Shenzhen, China) [26].

#### 2.5.4. Mechanical Properties

Mechanical parameters including the tensile strength (TS) and elongation at break (EAB) of the films were determined using a microcomputer-control electron universal testing machine (MTS Industrial Systems Co., Ltd., Shanghai, China). Before testing, all films were cut into samples of 2 cm × 7 cm. The initial grip distance and crosshead speed were set at 50 mm and 10 mm/min, respectively [3]. 

#### 2.5.5. Thermogravimetric Analysis

The thermal decomposition properties of the films were analyzed using a TG–DSC thermogravimetric analyzer (STA 449 F5, NETZSCH, Selb, Germany). Approximately 8 mg of the film sample was placed in an alumina crucible, while an empty crucible served as a blank control. In a N_2_ atmosphere, the temperature increased at a rate of 10 °C/min from 30 °C to 750 °C, and was used to analyze the thermal decomposition properties of the films [17].

#### 2.5.6. Water Contact Angle (WCA)

The WCA of the films was measured using a CA200 automatic optical contact angle measuring instrument (Guangdong North Precision Instrument Co., Ltd., Dongguan, China). Before measurement, the films were cut into a rectangle of 2 cm × 2 cm and placed on the horizontal moving platform of the WCA analyzer. Then, 5 μL of distilled water was dropped on the film surface using a syringe and the WCA was measured immediately from both sides by the SDC-200 (V3) software [27]. This was repeated three times and the average value was presented.

#### 2.5.7. Moisture Content (MC)

Films were cut into samples of 2 cm × 2 cm and weighed to obtain an initial weight (*M*_1_). Then, the films were dried at 105 °C until they reached a constant weight (*M*_2_). Moisture content was determined using the following formula [16]:

(2) $$MC\;\left(\%\right)=\frac{M_{1}-M_{2}}{M_{1}}\times 100$$

#### 2.5.8. Water Vapor Permeability (WVP)

The films were cut into circles with a diameter of 7.5 cm and placed into a test cup containing 8 mL of distilled water, which was then positioned in a water vapor transmission tester (Jinan Lan Guang Electromechanical Technology Co., Jinan, China) to obtain the WVP under the condition of 25 °C, 90% RH [28].

### 2.6. Functional Properties of Films 

#### 2.6.1. Light Transmittance and Opacity

A UV–VIS spectrophotometer was used to measure the optical properties of films. Films were cut into samples of 4.5 cm × 1.0 cm and vertically affixed to the inner side of the quartz cell. The light transmittance at 245 nm (*T*_245_), 300 nm (*T*_300_), 360 nm (*T*_360_), and absorbance at 600 nm (*A*_600_) were recorded using an empty quartz cell as the control. The opacity values of the film samples were calculated using Equation (3) [29]:

(3) $$Opacity=\frac{A_{600}}{d}$$

where *d* was the thickness (mm) of the film.

#### 2.6.2. Antioxidant Properties

The ABTS or DPPH radical scavenging capacity of films was measured according to the method in our previous work [21]. Films were dissolved in distilled water with a final concentration of 10 mg/mL. Then, 2 mL solution was mixed vigorously with 3.0 mL ABTS solution and allowed to stand for 6 min at 25 °C. Afterwards, the absorbance of the mixture was measured at 734 nm using the UV–VIS spectrophotometer. For the DPPH radical scavenging capacity, 2 mL film solution was added to 3 mL 0.1 mmol/L DPPH in ethanol. The mixture was mixed vigorously and allowed to stand in the dark at 25 °C for 20 min. Then, the absorbance of the mixture was measured at 517 nm using the UV–vis spectrophotometer. Ascorbic acid and purple sweet potato extract aqueous solution at 0.5 mg/mL were set as the controls. The radical scavenging rate was calculated using Equation (4):

(4) $$Radical\;Scavenging\;activity\;\left(\%\right)=\frac{\left(A_{c}-A_{t}\right)}{A_{c}}\times 100$$

where *A_c_* and *A_t_* are the absorbances of blank and tested film, respectively. 

### 2.7. Application of Films 

#### 2.7.1. Discoloration of PSC-PPE Films during Pork Spoilage

The test of monitoring pork spoilage was based on the method of Liu et al., with slight modifications [2]. The films were (diameter of 7.5 cm) fixed on the headspace of the Petri dish containing 20 g of fresh pork. Then, Petri dishes were sealed and kept at 4 °C or 25 °C. The color parameters of L*, a*, and b* for films were determined using a CS–200 precision colorimeter. The Δ*E* of the films was calculated according to Equation (1).

#### 2.7.2. Cherry Preservation

The cherries were immersed in film solution for 2 min, then stored in an incubator at 25 °C. Cherries without coating were used as a control. Photographs and the weight of the cherries were recorded on 1, 3, 5, 7, 9, and 11 days [30]. The following Equation (5) is used to calculate the change in weight loss over time:

(5) $$Weight\;loss\;(\%)=\frac{\left(W_{1}-W_{2}\right)}{W_{1}}\times 100$$

where *W*_1_ is the initially measured weight and *W*_2_ is the weight at subsequent days after.

### 2.8. Biodegradability of Films

The biodegradability of films was assessed based on their degradability in soil according to reference [31]. The films were weighed (*M*_1_) and buried in the soil at a depth of approximately 5 cm. Samples were retrieved every 3 days, dried at 105 °C for 4 h, and weighed (*M*_2_) to determine the biodegradability according to Equation (6), while the films were photographed.

(6) $$Biodegradability\;\left(\%\right)=\frac{M_{1}-M_{2}}{M_{1}}\times 100$$

### 2.9. Statistical Analysis

All the experiments were performed on at least three individual samples. Results were expressed as mean value ± standard deviation, and were analyzed using Origin 2021 (Origin Lab Corporation, Northampton, MA, USA). All statistical significance (*p* < 0.05) was analyzed using a one-way analysis of variance (ANOVA) with SPSS 24 software (SPSS Inc., Chicago, IL, USA). 

## 3. Results and Discussion

### 3.1. Characterization of Films

#### 3.1.1. Fourier Transform Infrared (FTIR) and X-ray Diffraction (XRD) Analysis

**Figure 1:**
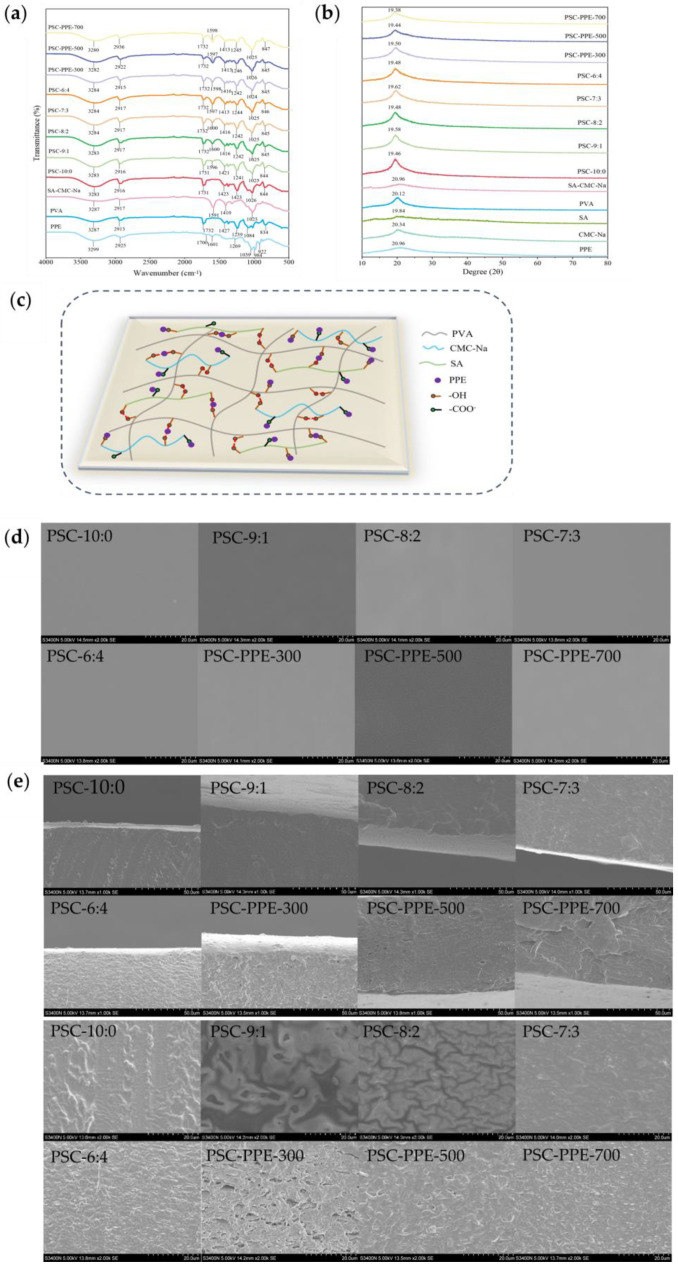

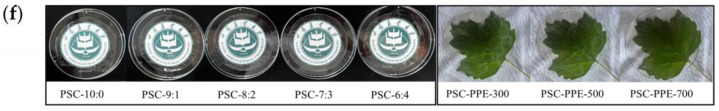
Spectral analysis of PSC-PPE films. FTIR spectra (**a**); XRD spectra (**b**); Schematic diagram of apparent intermolecular interactions between PPE and the PSC film matrix (**c**); SEM images of film surface (**d**); film cross-section (**e**); Visual appearance (**f**) of films, showing opacity and color.

FTIR spectra of the films were recorded (Figure 1a). The characteristic absorption peaks of PPE were observed at 3299 cm^−1^, 2925 cm^−1^, and 1601 cm^−1^ (O–H stretching, C–H stretching, and C=C stretching of the aromatic ring, respectively), and 1269 cm^−1^ and 1039 cm^−1^ (C–O stretching and C–H deformation of the aromatic ring, respectively) [32]. The characteristic absorption peaks of PVA were observed at 1084 cm^−1^ (O–H tensile vibration) and at 1427 cm^−1^ (bending vibration of CH–CH_2_), confirming the presence of the carbon skeleton of PVA [33]. The characteristic absorption peaks of SA/CMC-Na (without PVA) were observed at 2917 cm^−1^ (C–H stretching), 1591, 1410 cm^−1^ (–COO asymmetric and symmetric stretching, respectively), and 1023 cm^−1^ (C–O–C vibration) [20]. When PVA was combined with SA and CMC-Na, the O–H stretching peak slightly red shifted to 3287 cm^−1^, resulting from new intermolecular hydrogen bonds being formed between PVA and SA/CMC-Na [34]. The addition of PPE to PSC-6:4 induced a red shift of the O–H stretching peak from 3284 cm^−1^ to 3280 cm^−1^, suggesting the formation of hydrogen bonds between the PPE and the film matrix, which should increase the stability of the PPE. A similar hydrogen bond interaction was noted between SA and purple sweet potato peel extract [15].

The XRD spectrum of PPE exhibited a broad peak at 20.96°, indicating that the PPE was in an amorphous state (Figure 1b). The spectrum of SA powder had two diffraction peaks at 14.20 and 19.84°, respectively, consistent with a previous report [20]. The spectrum of SA/CMC-Na film had a diffraction peak at 20.96°, compared with 20.34° in pure CMC-Na and 19.84° in pure SA, indicating the formation of intermolecular interactions between SA and CMC-Na [20]. The diffraction peak intensities of films were slightly higher than those of the individual components, suggesting the formation of intermolecular hydrogen bonds in the films [22]. After addition to a film, the PPE diffraction peak disappeared, indicating that PPE was well dispersed in the film matrix. The proposed intermolecular interactions between PPE and the film matrix are shown schematically in Figure 1c. 

#### 3.1.2. Scanning Electron Microscopy (SEM) Analysis

The surface (Figure 1d) and cross-sectional (Figure 1e) morphology of films were observed using SEM. The film surfaces (Figure 1d) were smooth and continuous without visible granularity or phase separation. The pure PVA film (PSC-10:0) had a rough cross-section (Figure 1e), but with increasing SA/CMC-Na content, the cross-sectional morphology of the films became smoother with fine granularity at 6:4. In addition, relatively large voids were visible in the cross-section of PSC-PPE-300; the voids became smaller with increasing PPE addition, indicating a denser and more compact film structure with increasing PPE addition, which may contribute to the improved mechanical properties of the films. It appears that the regular film microstructure was disrupted by a small addition of PPE, weakening the interactions between the film components. With increasing PPE addition, cross-links were formed between the film components by PPE, resulting in a denser and more compact film microstructure. 

Similarly, chitosan film with 1–3 wt% rice berry anthocyanin extract added had a denser and more compact cross-sectional structure compared with pure chitosan film [35]. A rough cross-sectional microstructure was also observed for chitosan/chitin nanofiber films with added eggplant anthocyanins [36]. 

#### 3.1.3. Color and Opacity

Films without PPE were clear and colorless, but slightly pink with PPE present (Figure 1f). The color parameters of both PSC and PSC-PPE films were determined; with decreasing PVA concentration, the L* value of the PSC film decreased from 87.74 ± 0.30 (PSC-10:0) to 86.88 ± 0.33 (PSC-6:4) and the b* value increased from −2.79 ± 0.05 to −1.64 ± 0.15, which was attributed to the yellow hues of SA and CMC-Na (Table 2). In the presence of PPE, the L* value decreased from 86.88 ± 0.33 to 81.21 ± 0.07 and the a* value increased from −0.51 ± 0.04 to 2.86 ± 0.03 with increasing PPE concentration, consistent with the appearance of the PSC-PPE films (Figure 1f). Compared with PSC film, the total color difference (Δ*E*) value for PSC-PPE film increased markedly (*p* < 0.05). This is consistent with a previous report that the Δ*E* value of films based on PVA/cellulose nanocrystals increased with the addition of purple cabbage anthocyanin extract [3].

### 3.2. Performance Analysis of Films

#### 3.2.1. Color Response to pH Changes

**Figure 2:**
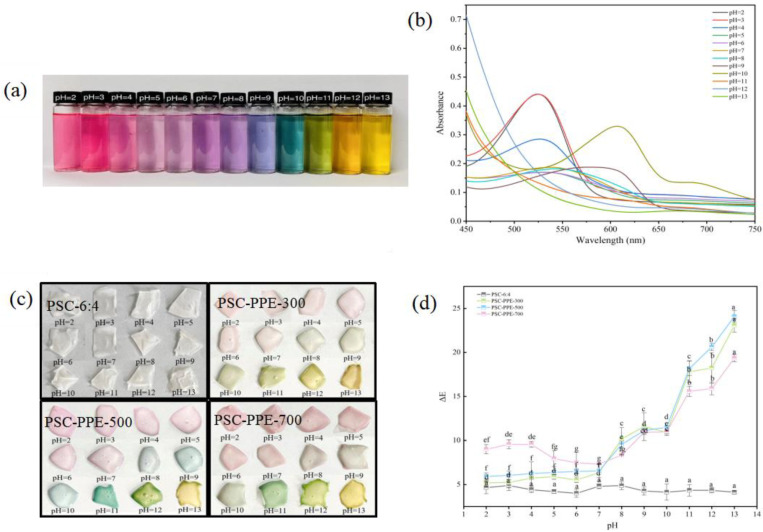
Visible color change (**a**) and UV–visible spectra (**b**) of purple sweet potato extract (PPE) over the pH range of 2.0–13.0. Visible color change (**c**) and total color difference (Δ*E*) (**d**) of films in the pH range of 2.0–13.0. Note: Values with different lowercase letters are significantly different (*p* < 0.05).

High sensitivity and a rapid response to pH changes are vital for the successful application of indicator films; the color changes of PPE solutions (Figure 2a) and changes in their UV–VIS absorption spectra (Figure 2b) were determined over the pH range of 2.0−13.0. At very acidic pH values, anthocyanins are in their most stable form of the flavylium cation with a positively charged pyrilium ring, which is a pink/red color [37]. The color of PPE solutions then became a lighter pink from pH 2.0 to 6.0, pale purple from pH 7.0 to 8.0, purple at pH 9.0, blue–green at pH 10.0, green at pH 11.0, orange at pH 12.0, and yellow at pH 13.0. Anthocyanins degrade in a strongly basic environment, changing to a chalcone structure [38]. The maximum absorption intensities of PPE decreased with increasing pH from acidic (pH 2.0) to neutral (pH 7.0; Figure 2b), similar to the color change of red cabbage anthocyanins [23]. In addition, the maximum absorption wavelength of PPE was 533 nm at pH 2.0 and 536 nm at pH 7.0. The red shift and reduced peak intensity in a mildly acidic or neutral environment (pH 4.0–7.0) results partly from spectral changes and partly from the degradation of the anthocyanin structure. In an alkaline environment (pH 9.0), degradation of PPE was much faster, consistent with a previous report [39].

The color change in response to the pH changes of PSC-PPE films was shifted relative to that of PPE solution. For example, the PPE solution was blue–green at pH 10.0, but PSC-PPE films were blue–green at pH 11.0 (Figure 2c). In addition, the PSC-PPE films had lighter colors than the PPE solution. The shift in color at a higher pH for the films suggests that the stability of PPE anthocyanins is improved in the PSC film environment. 

The change in Δ*E* of PSC-PPE films with pH was determined (Figure 2d). Generally, Δ*E* values > 5 are discernible to the naked eye and Δ*E* > 12 signifies a clearly visible color change [40,41]. All the Δ*E* values of films containing PPE were >5, indicating that both the PPE solution and PSC-PPE films are effective pH indicators over the entire pH range encountered in foods. 

#### 3.2.2. Thickness and Mechanical Properties

Thickness is an important characteristic for evaluating the physical properties of films, influencing their mechanical properties, water vapor transmittance, and light transmittance [42]. The thickness of PSC films decreased slightly with decreasing PVA content, indicating that the proportion of SA/CMC-Na has an insignificant effect on film thickness from 10:0 to 6:4 (Table 2). However, the thickness of PSC-6:4 film increased significantly with the addition of PPE at 500 and 700 mg/mL (*p* < 0.05), suggesting that a PPE content above 500 mg/mL results in major changes in the intermolecular interactions and the network structure of the film. Similarly, the thickness of cassava starch films increased with increased content of *Lycium ruthenicum* Murray berry anthocyanin extract [43].

**Figure 3:**
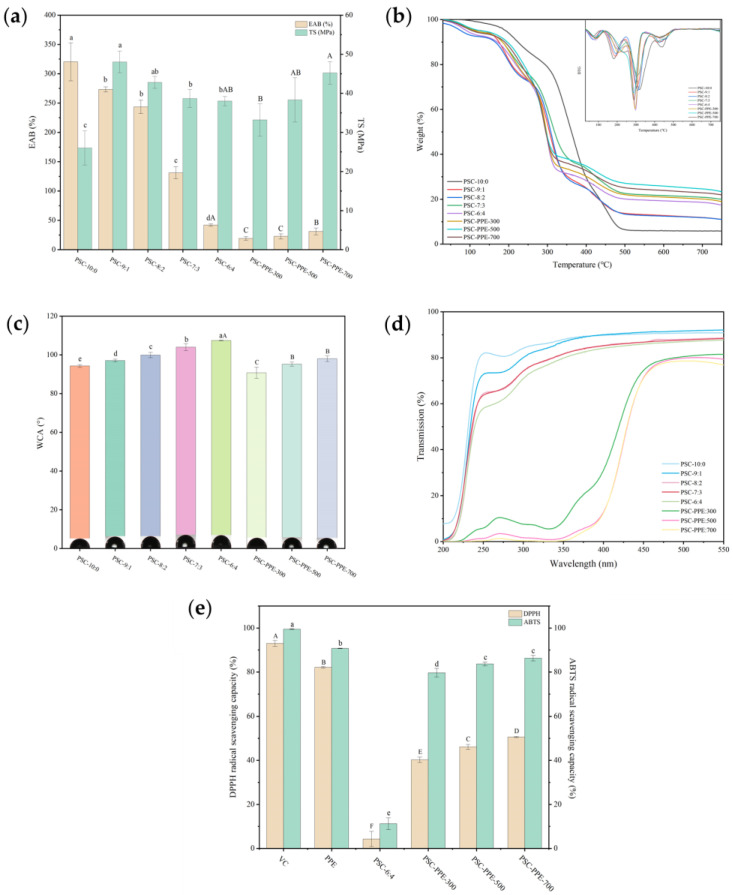
Mechanical and physicochemical properties of PSC films. Elongation at break (EAB) and tensile strength (TS) (**a**); TGA and DTG curves (**b**); Water contact angle (**c**); UV–visible light transmittance (**d**); DPPH and ABTS radical scavenging capacity compared with Vitamin C (VC) and purple sweet potato extract (PPE) (**e**). Note: Values with different lowercase letters are significantly different for PSC films without PPE; values with different capital letters are significantly different for PSC-6:4 films with different PPE concentrations (*p* < 0.05).

The tensile strength (TS) and elongation at break (EAB) reflect the mechanical resistance and flexibility of films. Pure PVA film (PSC-10:0) had the highest EAB, which decreased significantly with increasing addition of SA/CMC-Na (*p* < 0.05; Figure 3a). However, the TS of the films was improved by the addition of 700 mg PPE to the PSC-6:4 film, consistent with its greater thickness (Table 2). It appears that PPE molecules are involved in cross-links between polymer chains, which strengthens the film network structure and increases its TS. Similarly, the TS of sodium carboxymethyl starch film increased as the mulberry anthocyanin content increased from 0 to 5% [44].

Pure PVA film had the lowest TS, but its TS increased markedly with increasing addition of SA/CMC-Na, apparently because the formation of intermolecular hydrogen bonds between the –OH of PVA molecules and the –OH and –COOH of SA and CMC-Na chains reinforced the intermolecular interactions stabilizing the film network structure. The addition of PPE had no significant effect on the TS of the films.

### 3.3. Thermogravimetric Analysis of Films to Assess Stability

The thermogravimetric (TG) and differential thermogravimetric (DTG) curves of PSC films were determined using differential scanning calorimetry (Figure 3b). The TG curves of all the films exhibited four stages of weight loss. The first weight loss stage was observed from 50 to 100 °C, corresponding to the evaporation of adsorbed water from the films [18]. The second weight loss stage was observed between 150 and 200 °C, corresponding to the decomposition of glycerol [45]. The third stage, around 300 °C corresponds to polymer chain cleavage [18]. The fourth stage, between 400 and 500 °C, corresponds to the thermal decomposition of the film matrix. With the increasing addition of SA/CMC-Na, the temperature for maximum mass loss rate of the fourth degradation stage decreased and the final residue amount of the film increased. However, incorporation of PPE did not significantly change the thermal stability of the films, but only slightly increased the amount of final residue. Similarly, the addition of red cabbage anthocyanin extract did not alter the thermal stability of *Artemisia sphaerocephala* Krasch gum films [46].

### 3.4. Water Contact Angle, Moisture Content, and Water Vapor Permeability of Films 

The water contact angle (WCA) of the PSC films was determined (Figure 3c). The WCA of pure PVA film was 94.29 ± 0.71° and increased with decreasing PVA concentration. In addition, all WCAs were >90°, indicating that all the films have hydrophobic surfaces [27]. The addition of PPE decreased the WCA of the films compared with the PPE-free films (*p* < 0.05), indicating that the addition of PPE decreased the surface hydrophobicity of the films, but the increasing addition of PPE increased the WCA. 

The SEM images (Figure 1d,e) showed that the surfaces of all the films were smooth, whereas their cross-sections changed from smooth to granular with decreasing PVA content. The granular microstructure formed in PSC-6:4 appears to increase the water absorption of the film but decreases the wettability of its surface [47]. The addition of SA/CMC-Na generated hydrogen bonds between PVA and SA/CMC-Na, which is consistent with the increased WCA with decreasing PVA concentration. 

The polar groups (bulky aromatic and pyrylium rings) present in PPE anthocyanins appear to have affected the WCA. The SEM images (Figure 1) show that the granularity and porosity of the PSC-6:4 film increased significantly with the addition of PPE, increasing its hygroscopicity and decreasing its WCA. Similarly, the addition of cellulose nano crystals increased the WCA of gellan gum films [48]. However, with increasing PPE addition, the film porosity decreased, which would increase its surface hydrophobicity and, therefore, its WCA in agreement with a previous report [36]. 

Pure PVA film had the highest moisture content (MC; 18.43 ± 0.69%), which can be attributed to the hydrophilic groups in PVA, especially –OH (Table 2). With the increased addition of SA/CMC-Na, the MC of the films gradually decreased, apparently because of the hydrogen bonding between SA and CMC-Na blocking the accessibility of free hydroxyl groups to combine with water molecules. However, the MC of the film decreased slightly (*p* > 0.05) when PPE was added at 300–500 mg, apparently because of the more compact microstructure of PSC-PPE. Similarly, the addition of walnut peel extract decreased the MC of chitosan and guar gum films [49]. When 700 mg of PPE was added (PSC-PPE-700), the MC of the film markedly increased (*p* < 0.05) compared with PSC-PPE-300, apparently because of the former’s greater thickness. 

Packaging film with a low water vapor permeability (WVP) is desirable for food packaging applications [50]. The WVP of films without PPE decreased with decreasing PVA content (Table 2). The FTIR spectra (Figure 1a) indicated that hydrogen bonds were formed between hydrophilic groups on PVA and SA/CMC-Na, resulting in high water resistance [51]. Compared with PSC-6:4, the WVP of the film was higher after the addition of 300 mg of PPE, because of its more porous microstructure (Figure 1e). However, with the increasing PPE concentration, the WVP of the films decreased to 1.31 ± 0.12 × 10^−11^ g·cm/(cm^2^·s·Pa) for PSC-PPE-700 mg. The greater thickness and more compact structure of PSC-PPE-700 mg resulted in a lower WVP compared with other films. The water affinity of the film was also attenuated by the formation of hydrogen bonds between PPE and the polymer network [49]. In addition, the polar groups (bulky aromatic and pyrylium rings) present in PPE also affect the WVP [52]. Therefore, the addition of PPE at 500–700 mg improved the water resistance of PSC films. Similarly, the WVP of pullulan/chitosan/chitin nanofibers films was reduced by the addition of red cabbage extracts [53], as well as that of chitosan/chitin nanofiber films by eggplant anthocyanins [36].

### 3.5. Barrier Properties of Films

The light transmittance and opacity of the films is shown in Figure 3d and Table 2. PVA film had a high transparency since it was almost colorless and clear. As the PVA content decreased, the opacity of the films without PPE increased slightly (*p* < 0.05), i.e., increasing the SA/CMC-Na content reduced the visible light transmittance of the PVA films. Although the inclusion of PPE into the films changed the opacity insignificantly, it greatly decreased their UV–visible light transmittance (*p* < 0.05), apparently resulting from light absorption in the UV–visible wavelength range by PPE. Similarly, purple cabbage anthocyanins enhance the UV-blocking properties of films based on PVA/cellulose nanocrystals [3], and eggplant anthocyanins have the same effect on chitosan/chitin nanofiber films [36]. Hence, the UV radiation blocking capacity of PVA-SA/CMC-Na films was greatly improved by the addition of PPE, thereby minimizing UV-related discoloration, flavor, and nutrient loss in packaged food.

### 3.6. Antioxidant Properties of Films

Free radicals can cause the oxidative deterioration of food, so the antioxidant capacity of food packaging films is of great importance [29]. The antioxidant capacity of PSC-6:4 films, with or without PPE, was determined (Figure 3e). As expected, the film without PPE had little DPPH or ABTS scavenging capacity, but the capacity increased markedly (*p* < 0.05) with the addition of PPE. The antioxidant capacity of PPE has been reported previously [18]. Similarly, the addition of eggplant anthocyanins markedly increased the free radical scavenging capacity of chitosan/chitin nanofiber films [36].

### 3.7. Application of PSC-PPE Films for Pork and Cherry Preservation

**Figure 4:**
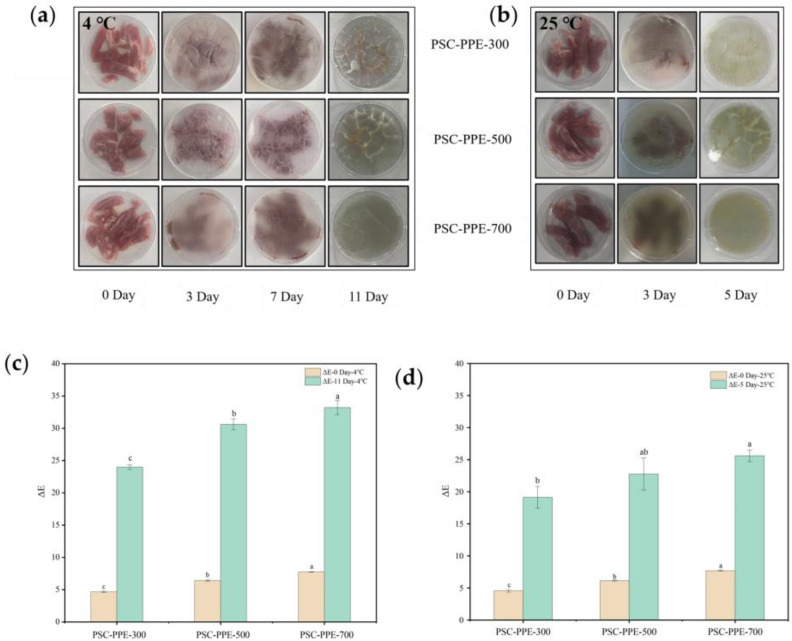

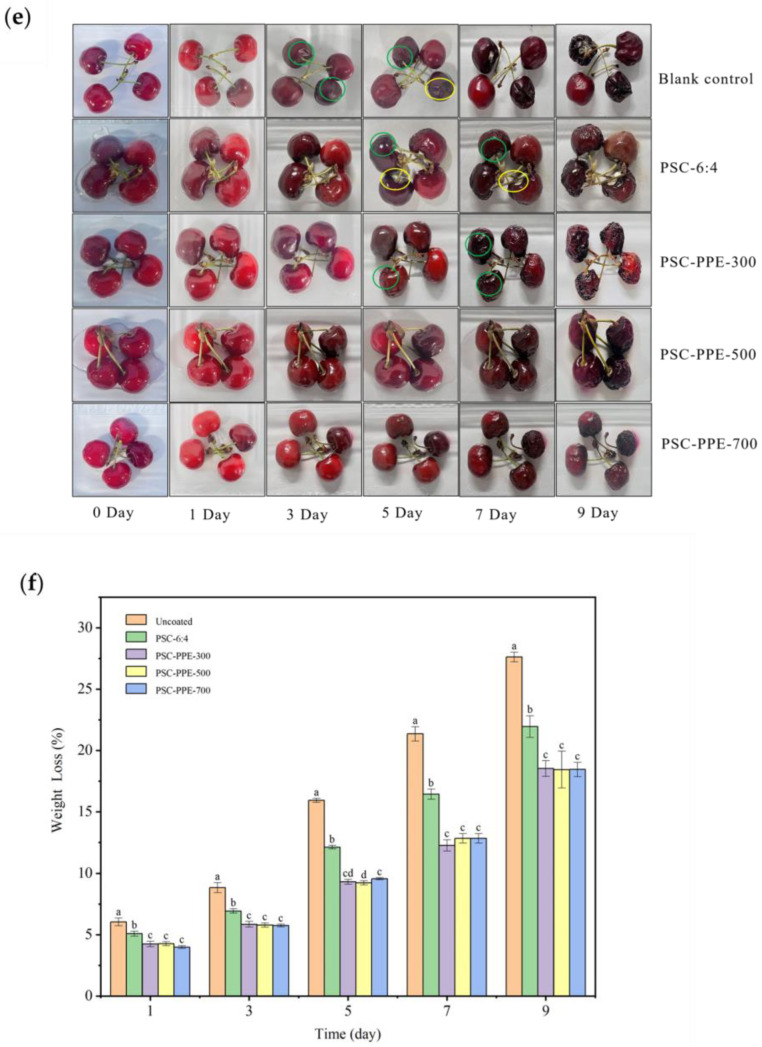
Color response of PSC-PPE films applied to pork during storage at 4 °C (**a**) and 25 °C (**b**). Total color difference (Δ*E*) of PSC-PPE films after storage for 11 days at 4 °C (**c**) and after 5 days at 25 °C (**d**). Appearance of uncoated cherries and those coated with PSC-6:4 or PSC-PPE film (**e**). Weight loss of uncoated and coated cherries as a function of time (**f**). Note: Values with different lowercase letters are significantly different at the same storage duration. The green and yellow circles in the figure indicate dehydration and mildew, respectively.

Fresh pork is highly susceptible to microbial infection and spoilage when exposed to air; microbial growth on the meat surface produces volatile nitrogenous compounds, a reduction in pH, and a color change in pH-indicator packaging [54]. The film color changes during the storage of pork were determined (Figure 4a,b). On the third day of storage at 25 °C, the films turned green, resulting from microbial decomposition of proteins to produce volatile nitrogenous compounds, increasing the pH. During storage at 4 °C, the green color change occurred at 11 days, indicating that the films have a sensitive response to volatile nitrogenous compounds. These compounds are basic and can induce the formation of anthocyanin carbinol bases, resulting in a color change in the film, similar to the color change of blueberry anthocyanins in starch/PVA films [55]. 

The *ΔE* of the films during pork storage is shown in Figure 4c,d. Δ*E* increased with an increasing PPE content, especially at 4 °C. As mentioned above, an Δ*E* > 5 is discernible to the naked eye; all Δ*E* values of PSC-PPE films were ≫5 at the end of the storage periods, indicating that the films are sensitive pH-indicators for monitoring pork freshness. This suggests that the PSC-PPE film has the potential to be developed as an indicator for monitoring meat freshness. 

Preservation of fruit from spoilage by mildew is another potential application for PSC-PPE films, so cherries were selected to evaluate the fruit preservation performance of PSC-PPE film coatings. The time taken for the appearance of mildew was compared (Figure 4e). Mildew appeared after 3 days on control untreated cherries, after 5 days for cherries coated with PSC film, and after 7 days on cherries coated with PSC-PPE-300. However, there was no visible mildew on cherries coated with PSC-PPE-500 and -700 after 9 days, but the cherries were dehydrated at this time. Meanwhile, the PSC-PPE coating decreased the dehydration of cherries (Figure 4f). The weight loss of uncoated cherries was 27.62 ± 0.40 after 9 days of storage, while the PPE coated cherries was below 19%. Clearly, cherries coated with PSC-PPE films had a markedly longer shelf-life and it appears that the barrier properties of the films and the antioxidant properties of PPE both contributed to this.

### 3.8. Biodegradability of PSC Films

**Figure 5:**
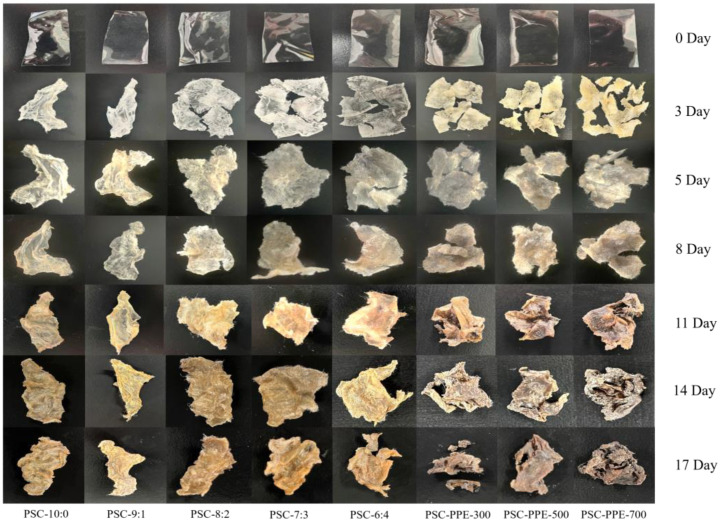
Appearance of PSC and PSC-PPE films after degradation in soil.

PSC and PSC-PPE films were buried in soil to assess their biodegradability in the environment (Figure 5, Table 3). After 3 days, soil moisture had migrated into the film samples, causing them to swell markedly and fragment. After 17 days, the PSC-6:4 and PSC-PPE films were more than 50% degraded (Table 3), suggesting that both films are fully biodegradable. The biodegradation rate of PSC-PPE-300 was significantly higher than that of PSC-6:4 film, apparently because of the much greater porosity of the former (Figure 1e). With an increasing PPE concentration, the biodegradation rate of the films decreased, apparently because of their less porous microstructure, but was still higher than that of PSC-6:4 film. It appears that the PSC-PPE films rapidly absorb moisture from the soil, thereby facilitating microbial invasion and degradation. Similarly, methylcellulose films degraded in soil faster with added *Syzygium cumini* skin anthocyanin extracts [56].

## 4. Conclusions

Functional pH indicator films were developed by combining PVA, SA, and CMC-Na, and incorporating purple sweet potato extract (PPE) as the pH indicator. The film with a PVA:SA/CMC-Na ratio of 6:4 had the highest water contact angle and acceptable mechanical properties, so it was chosen to determine the effect of adding PPE. PPE addition enhanced the intermolecular interactions between PVA and SA/CMC-Na, forming a compact microstructure and improving the mechanical properties of the film. The PSC-PPE films with 300–700 mg/g of PPE all exhibited a color change visible to the naked eye over the pH range of 2–13, and had higher ABTS and DPPH free radical scavenging capacities than PSC film without PPE. With 500 and 700 mg/g of PPE, the films had higher water barrier properties, higher UV-blocking properties, and were rapidly biodegraded in soil. The pH-sensitive films successfully indicated the freshness of stored pork and prolonged the shelf life of stored cherries. Currently, these findings indicate that PSC-PPE films have a potential use in active food packaging for meat and fruit. Future research should focus on the antibacterial activity of the films and broadening their application prospect in different food products such as seafood.

## Figures and Tables

**Table 1:** Composition of PSC and PSC-PPE films.

Films	PVA (g/100 mL)	SA (g/100 mL)	CMC-Na (g/100 mL)	PPE (mg/100 mL)
PSC-10:0	5.00	-	-	-
PSC-9:1	4.50	0.25	0.25	-
PSC-8:2	4.00	0.50	0.50	-
PSC-7:3	3.50	0.75	0.75	-
PSC-6:4	3.00	1.00	1.00	-
PSC-PPE-300	3.00	1.00	1.00	300
PSC-PPE-500	3.00	1.00	1.00	500
PSC-PPE-700	3.00	1.00	1.00	700

**Table 2:** Chromaticity, thickness, moisture content (MC), water vapor permeability (WVP), and barrier properties of films.

Films	PSC-10:0	PSC-9:1	PSC-8:2	PSC-7:3	PSC-6:4	PSC-PPE-300	PSC-PPE-500	PSC-PPE-700
L*	87.74 ± 0.30 ^a^	87.42 ± 0.06 ^ab^	87.27 ± 0.22 ^abc^	87.04 ± 0.16 ^bc^	86.88 ± 0.33 ^Ac^	83.87 ± 0.18 ^B^	82.64 ± 0.10 ^C^	81.21 ± 0.07 ^D^
a*	−0.37 ± 0.04 ^a^	−0.39 ± 0.06 ^a^	−0.41 ± 0.05 ^ab^	−0.48 ± 0.03 ^bc^	−0.51 ± 0.04 ^Dc^	0.93 ± 0.02 ^C^	2.32 ± 0.25 ^B^	2.86 ± 0.03 ^A^
b*	−2.79 ± 0.05 ^d^	−2.27 ± 0.09 ^c^	−2.03 ± 0.02 ^b^	−1.78 ± 0.07 ^a^	−1.64 ± 0.15 ^Ba^	−1.62 ± 0.10 ^C^	−1.75 ± 0.09 ^BC^	−1.34 ± 0.10 ^A^
Δ*E*	0.40 ± 0.19 ^d^	0.94 ± 0.09 ^c^	1.23 ± 0.15 ^bc^	1.57 ± 0.15 ^ab^	1.78 ± 0.32 ^Da^	4.55 ± 0.19 ^C^	6.13 ± 0.08 ^B^	7.71 ± 0.07 ^A^
Thickness (μm)	75.10 ± 7.12 ^a^	74.85 ± 4.76 ^a^	71.30 ± 3.82 ^ab^	69.10 ± 1.55 ^ab^	67.35 ± 1.17 ^Cb^	68.83 ± 0.79 ^C^	77.33 ± 0.63 ^B^	83.67 ± 0.34 ^A^
MC (%)	18.43 ± 0.69 ^a^	17.24 ± 0.28 ^a^	15.28 ± 1.33 ^b^	14.88 ± 0.67 ^b^	11.64 ± 0.58 ^ABc^	10.23 ± 0.46 ^B^	11.23 ± 0.97 ^AB^	12.63 ± 1.06 ^A^
WVP (×10^−11^ g.cm/(cm^2^·s·Pa))	20.56 ± 2.56 ^a^	18.30 ± 0.39 ^a^	15.07 ± 1.81 ^b^	13.03 ± 0.88 ^b^	5.11 ± 0.22 ^Bc^	7.76 ± 1.74 ^A^	3.51 ± 0.21 ^B^	1.31 ± 0.12 ^C^
*T*_245 nm_	78.23 ± 1.35 ^a^	71.05 ± 0.55 ^b^	62.67 ± 0.84 ^c^	61.99 ± 0.37 ^c^	54.48 ± 1.32 ^Ad^	4.37 ± 1.11 ^B^	1.10 ± 1.40 ^C^	0.21 ± 1.68 ^C^
*T*_300 nm_	82.17 ± 1.70 ^a^	80.55 ± 0.62 ^a^	74.78 ± 0.55 ^b^	74.36 ± 1.21 ^b^	69.50 ± 1.30 ^Ac^	6.11 ± 0.23 ^B^	1.44 ± 0.29 ^C^	0.28 ± 0.92 ^C^
*T*_360n m_	87.60 ± 1.16 ^a^	87.46 ± 0.58 ^a^	83.11 ± 0.47 ^b^	82.01 ± 0.80 ^bc^	81.28 ± 1.02 ^Ac^	13.49 ± 0.07 ^B^	4.09 ± 0.05 ^C^	1.41 ± 0.14 ^D^
Opacity	1.23 ± 0.11 ^c^	1.46 ± 0.15 ^bc^	1.58 ± 0.02 ^ab^	1.73 ± 0.06 ^a^	1.79 ± 0.06 ^Aa^	1.55 ± 0.01 ^B^	1.62 ± 0.01 ^B^	1.90 ± 0.05 ^A^

Note: Values with different lowercase letters are significantly different for PSC films without PPE; values with different capital letters are significantly different for PSC-6:4 films with different PPE concentrations (*p* < 0.05).

**Table 3:** Biodegradability of films in soil.

Films	1 Day	5 Days	8 Days	11 Days	14 Days	17 Days
PSC-10:0	1.36 ± 0.20 ^f^	5.99 ± 0.31 ^f^	9.26 ± 0.38 ^e^	10.18 ± 0.66 ^g^	10.86 ± 0.27 ^h^	13.35 ± 0.48 ^g^
PSC-9:1	2.63 ± 0.15 ^de^	7.48 ± 0.33 ^f^	11.88 ± 0.49 ^e^	20.67 ± 1.34 ^f^	21.09 ± 1.47 ^g^	24.38 ± 1.78 ^f^
PSC-8:2	2.62 ± 0.23 ^de^	16.29 ± 1.85 ^e^	23.49 ± 1.34 ^d^	27.15 ± 1.56 ^e^	29.20 ± 0.94 ^f^	31.29 ± 1.07 ^e^
PSC-7:3	3.84 ± 0.39 ^cd^	22.44 ± 0.94 ^d^	30.24 ± 2.44 ^c^	38.82 ± 1.29 ^d^	42.70 ± 1.80 ^e^	45.31 ± 0.85 ^d^
PSC-6:4	6.30 ± 0.24 ^a^	26.68 ± 0.56 ^c^	37.60 ± 1.35 ^b^	50.26 ± 1.87 ^c^	52.3 ± 1.95 ^d^	52.47 ± 1.12 ^c^
PSC-PPE-300	4.87 ± 0.47 ^b^	38.60 ± 3.25 ^a^	45.06 ± 1.87 ^a^	60.81 ± 0.48 ^a^	63.66 ± 0.70 ^a^	64.29 ± 1.75 ^a^
PSC-PPE-500	3.09 ± 0.68 ^cd^	40.53 ± 1.17 ^a^	43.12 ± 2.11 ^a^	57.17 ± 1.34 ^b^	58.44 ± 1.20 ^b^	60.22 ± 1.05 ^b^
PSC-PPE-700	1.88 ± 0.47 ^ef^	34.49 ± 2.74 ^b^	38.33 ± 0.98 ^b^	51.52 ± 3.03 ^c^	55.36 ± 2.06 ^c^	58.18 ± 0.95 ^b^

Note: Values with different lowercase letters are significantly different (*p* < 0.05).

## Data Availability

Data will be made available on reasonable request.

## References

[1] Koshy R.R., Koshy J.T., Mary S.K., Sadanandhan S., Pothan L.A. (2021). Preparation of pH sensitive film based on starch/carbon nano dots incorporating anthocyanin for monitoring spoilage of pork. Food Control.

[2] Liu J., Huang J., Ying Y., Hu L., Hu Y. (2021). pH-sensitive and antibacterial films developed by incorporating anthocyanins extracted from purple potato or roselle into chitosan/polyvinyl alcohol/nano-ZnO matrix: Comparative study. Int. J. Biol. Macromol..

[3] He Y., Lu L., Lin Y., Li R., Yuan Y., Lu X., Zou Y., Zhou W., Wang Z., Li J. (2022). Intelligent pH-sensing film based on polyvinyl alcohol/cellulose nanocrystal with purple cabbage anthocyanins for visually monitoring shrimp freshness. Int. J. Biol. Macromol..

[4] Zhang J., Zhang J., Huang X., Arslan M., Shi J., Li Z., Gong Y., Holmes M., Zou X. (2023). Fabrication and characterization of polyvinyl alcohol/sodium alginate/zein/chitosan bilayer film for dynamic visualization of pork quality. Int. J. Biol. Macromol..

[5] Wang Y., Zhang J., Zhang L. (2022). An active and pH-responsive film developed by sodium carboxymethyl cellulose/polyvinyl alcohol doped with rose anthocyanin extracts. Food Chem..

[6] Miranda B.M., Cruz M.V., De Campos I.T.N., Fernandes K.F., Silva F.A. (2022). A halochromic film containing *Plinia cauliflora* peel anthocyanins loaded into a cashew gum polysaccharide-polyvinyl alcohol matrix. Waste Biomass Valorization.

[7] He Y., Li B., Du J., Cao S., Liu M., Li X., Ren D., Wu X., Xu D. (2022). Development of pH-responsive absorbent pad based on polyvinyl alcohol/agarose/anthocyanins for meat packaging and freshness indication. Int. J. Biol. Macromol..

[8] Zhang J., Huang X., Shi J., Liu L., Zhang X., Zou X., Xiao J., Zhai X., Zhang D., Li Y. (2021). A visual bi-layer indicator based on roselle anthocyanins with high hydrophobic property for monitoring griskin freshness. Food Chem..

[9] Liu D., Zhang C., Pu Y., Chen S., Li H., Zhong Y. (2023). Novel colorimetric films based on polyvinyl alcohol/sodium carboxymethyl cellulose doped with anthocyanins and betacyanins to monitor pork freshness. Food Chem..

[10] Yam K.L., Takhistov P.T., Miltz J. (2010). Intelligent packaging: Concepts and applications. J. Food Sci..

[11] Subramanian K., Logaraj H., Ramesh V., Mani M., Balakrishnan K., Selvaraj H., Pugazhvendan S.R., Velmurugan S., Aruni W. (2022). Intelligent pH indicative film from plant-based extract for active biodegradable smart food packing. J. Nanomater..

[12] Akhila K., Sultana A., Ramakanth D., Gaikwad K.K. (2023). Monitoring freshness of chicken using intelligent pH indicator packaging film composed of polyvinyl alcohol/guar gum integrated with Ipomoea coccinea extract. Food Biosci..

[13] Priyadarshi R., Ezati P., Rhim J.W. (2021). Recent advances in intelligent food packaging applications using natural food colorants. Food Sci. Technol..

[14] Wang X., Yong H., Gao L., Li L., Jin M., Liu J. (2018). Preparation and characterization of antioxidant and pH-sensitive films based on chitosan and black soybean seed coat extract. Food Hydrocoll..

[15] Zhao M., Nuerjiang M., Bai X., Feng J., Kong B., Sun F., Li Y., Xia X. (2022). Monitoring dynamic changes in chicken freshness at 4 °C and 25 °C using pH-sensitive intelligent films based on sodium alginate and purple sweet potato peel extracts. Int. J. Biol. Macromol..

[16] Huang J., Chen M., Zhou Y., Li Y., Hu Y. (2020). Functional characteristics improvement by structural modification of hydroxypropyl methylcellulose modified polyvinyl alcohol films incorporating roselle anthocyanins for shrimp freshness monitoring. Int. J. Biol. Macromol..

[17] Zheng T., Tang P., Li G. (2023). Development of a pH-sensitive film based on collagen/chitosan/ZnO nanoparticles and mulberry extract for pork freshness monitoring. Food Chem..

[18] Dong S., Zhang Y., Lu D., Gao W., Zhao Q., Shi X. (2023). Multifunctional intelligent film integrated with purple sweet potato anthocyanin and quercetin-loaded chitosan nanoparticles for monitoring and maintaining freshness of shrimp. Food Packag. Shelf Life.

[19] Yong H., Liu J. (2020). Recent advances in the preparation, physical and functional properties, and applications of anthocyanins-based active and intelligent packaging films. Food Packag. Shelf Life.

[20] Yang Y., Yu X., Zhu Y., Zeng Y., Fang C., Liu Y., Hu S., Ge Y., Jiang W. (2022). Preparation and application of a colorimetric film based on sodium alginate/sodium carboxymethyl cellulose incorporated with rose anthocyanins. Food Chem..

[21] Qin J., Yang M., Wang Y., Wa W., Zheng J. (2021). Interaction between caffeic acid/caffeic acid phenethyl ester and micellar casein. Food Chem..

[22] Wang Y., Yang M., Qin J., Wa W. (2021). Interactions between puerarin/daidzein and micellar casein. J. Food Biochem..

[23] Liu D., Cui Z., Shang M., Zhong Y. (2021). A colorimetric film based on polyvinyl alcohol/sodium carboxymethyl cellulose incorporated with red cabbage anthocyanin for monitoring pork freshness. Food Packag. Shelf Life.

[24] Zhou X., Yu X., Xie F., Fan Y., Xu X., Qi J., Xiong G., Gao X., Zhang F. (2021). pH-responsive double-layer indicator films based on konjac glucomannan/camellia oil and carrageenan/anthocyanin/curcumin for monitoring meat freshness. Food Hydrocoll..

[25] Yong H., Wang X., Bai R., Miao Z., Zhang X., Liu J. (2019). Development of antioxidant and intelligent pH-sensing packaging films by incorporating purple-fleshed sweet potato extract into chitosan matrix. Food Hydrocoll..

[26] Valencia G.A., Luciano C.G., Lourenço R.V., Bittante A.M.Q.B., Do Amaral Sobral P.J. (2019). Morphological and physical properties of nano-biocomposite films based on collagen loaded with laponite^®^. Food Packag. Shelf Life.

[27] Valencia G.A., Luciano C.G., Lourenço R.V., Do Amaral Sobral P.J. (2018). Microstructure and physical properties of nano-biocomposite films based on cassava starch and laponite. Int. J. Biol. Macromol..

[28] Guo H., Shao C., Ma Y., Zhang Y., Lu P. (2023). Development of active and intelligent pH food packaging composite films incorporated with litchi shell extract as an indicator. Int. J. Biol. Macromol..

[29] Shan P., Wang K., Yu F., Yi L., Sun L., Li H. (2023). Gelatin/sodium alginate multilayer composite film crosslinked with green tea extract for active food packaging application. Colloid Surface A.

[30] Zhao S., Jia R., Yang J., Dai L., Ji N., Xiong L., Sun Q. (2022). Development of chitosan/tannic acid/corn starch multifunctional bilayer smart films as pH-responsive actuators and for fruit preservation. Int. J. Biol. Macromol..

[31] Jiang H., Zhang W., Cao J., Jiang W. (2022). Effect of purple sugarcane peel extracts on properties of films based on lemon peel waste pectin and the application in the visible detection of food freshness. Food Hydrocoll..

[32] Zong Z., Liu M., Chen H., Farag M.A., Wu W., Fang X., Niu B., Gao H. (2023). Preparation and characterization of a novel intelligent starch/gelatin binary film containing purple sweet potato anthocyanins for *Flammulina velutipes* mushroom freshness monitoring. Food Chem..

[33] Pereira V.A., de Arruda I.N.Q., Stefani R. (2014). Active Chitosan/PVA films with anthocyanins from *Brassica oleraceae* (red cabbage) as time-temperature indicators for application in intelligent food packaging. Food Hydrocoll..

[34] Zhai X., Shi J., Zou X., Wang S., Jiang C., Zhang J., Huang X., Zhang W., Holmes M. (2017). Novel colorimetric films based on starch/polyvinyl alcohol incorporated with roselle anthocyanins for fish freshness monitoring. Food Hydrocoll..

[35] Eze F.N., Jayeoye T.J., Singh S. (2022). Fabrication of intelligent pH-sensing films with antioxidant potential for monitoring shrimp freshness via the fortification of chitosan matrix with broken Riceberry phenolic extract. Food Chem..

[36] Wang F., Xie C., Tang H., Hao W., Wu J., Sun Y., Liu Y., Jiang L. (2023). Development, characterization and application of intelligent/active packaging of chitosan/chitin nanofibers films containing eggplant anthocyanins. Food Hydrocoll..

[37] Calderaro A., Barreca D., Bellocco E., Smeriglio A., Trombetta D., Laganà G. (2020). Colored phytonutrients: Role and applications in the functional foods of anthocyanins. Phytonutrients in Food.

[38] Grajeda-Iglesias C., Figueroa-Espinoza M.C., Barouh N., Barea B., Fernandes A., de Freitas V., Salas E. (2016). Isolation and characterization of anthocyanins from *Hibiscus sabdariffa* flowers. J. Nat. Prod..

[39] Zhang R., Ye S., Guo Y., Wu M., Jiang S., He J. (2023). Studies on the interaction between homological proteins and anthocyanins from purple sweet potato (PSP): Structural characterization, binding mechanism and stability. Food Chem..

[40] Moradi M., Tajik H., Almasi H., Forough M., Ezati P. (2019). A novel pH-sensing indicator based on bacterial cellulose nanofibers and black carrot anthocyanins for monitoring fish freshness. Carbohydr. Polym..

[41] Jamroz E., Kulawik P., Guzik P., Duda I. (2019). The verification of intelligent properties of furcellaran films with plant extracts on the stored fresh Atlantic mackerel during storage at 2 °C. Food Hydrocoll..

[42] Zhang K., Huang T.S., Yan H., Hu X., Ren T. (2019). Novel pH-sensitive films based on starch/polyvinyl alcohol and food anthocyanins as a visual indicator of shrimp deterioration. Int. J. Biol. Macromol..

[43] Qin Y., Liu Y., Yong H., Liu J., Liu J. (2019). Preparation and characterization of active and intelligent packaging films based on cassava starch and anthocyanins from *Lycium ruthenicum* Murr. Int. J. Biol. Macromol..

[44] Zhang C., Sun G., Cao L., Wang L. (2020). Accurately intelligent film made from sodium carboxymethyl starch/κ-carrageenan reinforced by mulberry anthocyanins as an indicator. Food Hydrocoll..

[45] Gutiérrez T.J., Alvarez V.A. (2018). Bionanocomposite films developed from corn starch and natural and modified nano-clays with or without added blueberry extract. Food Hydrocoll..

[46] Liang T., Sun G., Cao L., Li J., Wang L. (2018). A pH and NH_3_ sensing intelligent film based on *Artemisia sphaerocephala* Krasch. gum and red cabbage anthocyanins anchored by carboxymethyl cellulose sodium added as a host complex. Food Hydrocoll..

[47] Saleem Akhtar H.M., Zhao Y., Li L., Shi Q. (2024). Novel active composite films based on carboxymethyl cellulose and sodium alginate incorporated with phycocyanin: Physico-chemical, microstructural and antioxidant properties. Food Hydrocoll..

[48] Yue C., Huang Y., Kong B., Wang G. (2022). Effect of anthocyanin indicator addition on cellulose nanocrystals/gellan gum-based intelligent packaging films. Membranes.

[49] Jiang L., Wang F., Xie X., Xie C., Li A., Xia N., Gong X., Zhang H. (2022). Development and characterization of chitosan/guar gum active packaging containing walnut green husk extract and its application on fresh-cut apple preservation. Int. J. Biol. Macromol..

[50] Gasti T., Dixit S., Hiremani V.D., Chougale R.B., Masti S.P., Vootle S.K., Mudigoudra B.S. (2021). Chitosan/pullulan based films incorporated with clove essential oil loaded chitosan-ZnO hybrid nanoparticles for active food packaging. Carbohydr. Polym..

[51] Alizadeh-Sani M., Tavassoli M., Mohammadian E., Ehsani A., Khaniki G.J., Priyadarshi R., Rhim J.W. (2020). pH-responsive color indicator films based on methylcellulose/chitosan nanofiber and barberry anthocyanins for real-time monitoring of meat freshness. Int. J. Biol. Macromol..

[52] Zhou Q., Chen J., Wang C., Yang G., Janaswamy S., Xu F., Liu Z. (2022). Preparation and characterization of lignin nanoparticles and chitin nanofibers reinforced PVA films with UV shielding properties. Ind. Crops Prod..

[53] Wu C., Jiang H., Zhao J., Humayun M., Wu S., Wang C., Zhi Z., Pang J. (2022). A novel strategy to formulate edible active-intelligent packaging films for achieving dynamic visualization of product freshness. Food Hydrocoll..

[54] Hao Y., Kang J., Guo X., Sun M., Li H., Bai H., Cui H., Shi L. (2023). pH-responsive chitosan-based film containing oregano essential oil and black rice bran anthocyanin for preserving pork and monitoring freshness. Food Chem..

[55] Wu W., Liu L., Zhou Y., Shao P. (2024). Highly ammonia-responsive starch/PVA film with gas absorption system as the ‘bridge’ for visually spoilage monitoring of animal-derived food. Food Chem..

[56] Filipini G.D.S., Romani V.P.R., Martins V.G. (2020). Biodegradable and active-intelligent films based on methylcellulose and jambolão (*Syzygium cumini*) skins extract for food packaging. Food Hydrocoll..

